# Prevalence, severity and risk factors for asthma in school-going adolescents in KwaZulu Natal, South Africa

**DOI:** 10.1136/bmjresp-2022-001498

**Published:** 2023-05-16

**Authors:** Reratilwe Mphahlele, Maia Lesosky, Refiloe Masekela

**Affiliations:** 1 Paediatrics and Child Health, University of KwaZulu Natal, Durban, KwaZulu Natal, South Africa; 2 Division of Epidemiology and Biostatistics, University of Cape Town, Rondebosch, South Africa; 3 Global Health Trials Unit, Liverpool School of Tropical Medicine, Liverpool, UK

**Keywords:** Asthma, Asthma Epidemiology, Inhaler devices, Paediatric asthma, Asthma in primary care

## Abstract

**Background:**

Asthma remains highly prevalent, with more severe symptoms in low-income to middle-income countries (LMICs) compared with high-income countries. Identifying risk factors for severe asthma symptoms can assist with improving outcomes. We aimed to determine the prevalence, severity and risk factors for asthma in adolescents in an LMIC.

**Methods:**

A cross-sectional survey using the Global Asthma Network written and video questionnaires was conducted in adolescents aged 13 and 14 from randomly selected schools in Durban, South Africa, between May 2019 and June 2021.

**Results:**

A total of 3957 adolescents (51.9% female) were included. The prevalence of lifetime, current and severe asthma was 24.6%, 13.7% and 9.1%, respectively. Of those with current and severe asthma symptoms; 38.9% (n=211/543) and 40.7% (n=147/361) had doctor-diagnosed asthma; of these, 72.0% (n=152/211) and 70.7% (n=104/147), respectively, reported using inhaled medication in the last 12 months. Short-acting beta agonists (80.4%) were more commonly used than inhaled corticosteroids (13.7%). Severe asthma was associated with: fee-paying school quintile (adjusted OR (CI)): 1.78 (1.27 to 2.48), overweight (1.60 (1.15 to 2.22)), exposure to traffic pollution (1.42 (1.11 to 1.82)), tobacco smoking (2.06 (1.15 to 3.68)), rhinoconjunctivitis (3.62 (2.80 to 4.67)) and eczema (2.24 (1.59 to 3.14)), all p<0.01.

**Conclusion:**

Asthma prevalence in this population (13.7%) is higher than the global average (10.4%). Although common, severe asthma symptoms are underdiagnosed and associated with atopy, environmental and lifestyle factors. Equitable access to affordable essential controller inhaled medicines addressing the disproportionate burden of asthma is needed in this setting.

**What is already known on this topic**

- There is an increasing prevalence and difference in risk factors for asthma in children and adolescents in low-income and middle-income countries (LMICs) compared with high-income countries.

**What this study adds**

- Asthma is poorly diagnosed in adolescents in LMICs, and access to essential inhaled controller medicines is limited in those diagnosed. Severe asthma symptoms, although common, are underappreciated. Unique and increasingly common environmental and lifestyle risk factors contribute to severe asthma.

**How this study might affect research, practice or policy**

- The results of this study can inform regional and national solutions for health systems in LMICs for reducing risk factors, focusing on asthma education, maintaining long-term follow-up and issuing appropriate inhaled asthma medication.

## Introduction

Asthma, the most common chronic respiratory disease (CRD) among children globally, affected an estimated 262 million people by 2019 and remains one of the most common CRDs across the life course.^1 2^ Although largely preventable, almost all asthma-related deaths occur in low-income and middle-income countries (LMICs), where underdiagnosis, suboptimal treatment and research infrastructure remain challenging.^3^ The WHO has identified asthma as a hidden source of poverty in LMICs, affecting economic and social development.^4^

The International Study of Asthma and Allergies in Childhood (ISAAC) is the most extensive questionnaire-based epidemiologic study worldwide and provides data on the prevalence, trends and potential risk factors of asthma in children and adolescents.^5^ Following ISAAC, the Global Asthma Network (GAN) phase I study assessed worldwide asthma trends over three decades in children aged 6–7 and adolescents aged 13–14.^5^ The prevalence of current wheeze in adolescents ranged from 0.9% (New Delhi, India) to 21.3% (Cape Town, South Africa), with a mean of 10.4%. Furthermore, the prevalence of current wheeze remained stable in high-income countries (HICs), increased in LMICs, but dropped in low-income countries.^5^

In Africa, a much larger proportion of adolescents (15.4%) than the global average (10.4%) suffer from current asthma symptoms.^5^ ISAAC, GAN and other African data highlight the increasing prevalence of asthma in urban school children and a rural-urban gradient with a higher reported prevalence of current wheeze in urban than rural populations.^5 6^ While the magnitude of change has declined over the last three decades, the proportion of those who report severe asthma symptoms is increasing, with only a third of those with asthma symptoms having ever been diagnosed with asthma.^7^ Factors responsible for the greater increase of asthma severity in LMICs, including in South Africa, are diverse and evolving and may not be comparable to those in HICs. As the prevalence and severity of asthma improve in HICs, the role of socioeconomic determinants of health and poor access to affordable quality-assured medication in LMIC requires investigation.^2^

Variability in the prevalence of asthma across different geographical areas suggests that environmental factors may play a role. Modifiable risk factors identified in LMICs, including smoking, outdoor air pollution and dietary changes, are thought to be primarily influenced by the increasing rate and degree of urbanisation. Air pollution, particularly exposure to traffic, has been associated with increased asthma, rhinitis and eczema symptoms.^8^ Systematic reviews are beginning to report the effect of diet on airway microbiota and immune response.^9^ In urban areas, a higher prevalence of food insecurity aggravated by poverty and unemployment has been shown to impact the affordability of essential foods such as fruits and vegetables.^10^ A western diet (low in fruits and vegetables and high in refined grains) is thought to promote a proinflammatory environment. In contrast, a Mediterranean diet (high in fruits and vegetables and low in refined grains) promotes an anti-inflammatory environment.^9^ Despite the double burden of malnutrition in LMICs, overweight and obesity due to a poor diet are widespread and associated with asthma prevalence and severity.^11^

Given this context, we first aimed to determine the prevalence of asthma and severe asthma in school-going adolescents using the standardised GAN methodology in South Africa. Second, we aimed to explore factors associated with asthma in South Africa, including diet’s impact on asthma outcomes.

## Methods

### Study design and population

A cross-sectional survey was conducted using the GAN written and video questionnaire in school-going adolescents between the ages of 13 and 14 years. The English questionnaire was back-translated into isiZulu. Demographic data included date of birth, school, sex, weight and height. Environmental questions included exercise, traffic exposure, pets and siblingship. Lifestyle questions included television watching, diet, tobacco smoking and computer use (encompassing electronics/internet use including PlayStation, smartphone, tablet, Chat, Facebook, games, Twitter and YouTube).^12^ Adolescents whose parents/guardians signed consent and assented were included in the study.

### Setting

The study period was between May 2019 and June 2021 in primary and secondary schools in Durban (urban) and Richards Bay (rural), KwaZulu Natal (KZN), the second-most populous province in South Africa, with 11 065 240 residents.^13^ KZN has the largest child population in South Africa, at 2.6 million, with 62% of its child population being classified as rural compared with Western Cape and Gauteng, with 94% and 97% of its child population being urban-based.^13 14^

Durban is the largest city in KZN, with 13% of the population living in informal settlements in urban areas.^13^ Richards Bay is a secondary city that lies on the northeast coast of KZN and is classified as a rural area as the population density is less than 500 per km^2^.^13^

The Department of Basic Education (DoBE) has a school ranking system with a quintile based on the school’s socioeconomic status (SES) and is determined by measures of average income, unemployment rate and general literacy level in the school’s geographical area.^15^ Quintiles range from 1 to 5, where schools from the poorest geographical areas are categorised in quintiles 1–3 and classified as non-fee-paying on the assumption that parents cannot afford fees. Quintiles 4 to 5 are classified as fee paying schools from wealthier geographical areas where parents can afford fees.^15^ The quintiles were used as a proxy for SES.

### Recruitment

Schools were randomly selected from the DoBE school’s database, where 6025 schools are stratified by school phase: primary (64.1%), secondary (26.9%) and combined schools (9.0%). Selected schools were sequentially approached to participate in the study by telephone, email or visit until the required sample size was reached. Grades with most children aged 13–14 were used to recruit participants.

### Sample size and power

As per GAN methodology, a sample size of 3000 participants is required to detect yearly changes of at least 0.6% after 5 years with a power of 90% at the 5% significance level when the current prevalence is 20%.^12^ Cluster sampling was used with each school as a cluster. This study aimed to enrol 3500 participants, with an additional 500 to address a 10% attrition rate for non-response and data entry errors.

### Outcomes

All outcomes were defined using the ISAAC/GAN methodology. *Lifetime asthma* was defined as ever having a wheeze. *Current asthma* (asthma symptom prevalence) was defined as experiencing wheezing in the last 12 months.^12^ *Severe asthma* was defined by a positive answer to any of the following symptoms in the last 12 months: four or more attacks of wheezing, sleep disturbed due to wheezing one or more nights a week and wheezing severe enough to limit speech.^12^ Rhinoconjunctivitis was defined as sneezing, runny or blocked nose accompanied by itchy, watery eyes without a cold or the influenza in the last 12 months.^12^ Eczema was described as an itchy rash at any time during the previous 12 months affecting the folds of the elbows, behind the knees, ankles, under the buttocks, neck, ears or eyes for at least 6 months.^12^ *Inhaled medicines use* was defined as asthma pump/spray used in the past 12 months. *Oral medicines* included prednisone, short-acting beta-agonists (SABA), theophylline and mast cell stabilisers. The frequency of use of drugs was categorised into ‘when needed’, ‘short courses’ and ‘daily’ in the past 12 months. *Asthma outcome indicators* were defined by at least one urgent doctor or emergency room (ER) visit or hospitalisation, at least one school day missed due to breathing problems, cough at night and exercise-induced asthma in the past 12 months. *Exercise-induced asthma* was defined by a wheezy chest during or after exercise.

### Exposures

*Traffic pollution* was defined as self-reported truck frequency outside the respondent’s home. ‘Frequently or almost the whole day’ was defined as exposure to traffic pollution.^7^ Other environmental variables were having *pets* at home (cat and/or dog), exercise *recommended by WHO* as engaging in≥3 physical activities weekly^16^ and a *sedentary lifestyle* as ≥5 hours of daily television watching or computer use. *Paracetamol use* was at least once a month use as opposed to once or less in a year. *Current tobacco smoking and other types of smoking* (eg, hubbly-bubbly, vaping, crack pipe) were assessed. *Diet* was determined by the frequency of consumption of meat, seafood, fruit, cooked vegetables, raw vegetables, burgers, fast foods and fizzy drinks in the past 12 months. The *Mediterranean* and *Western diets* were determined using a modified score pattern developed by Nagel *et al*.^17^ Body mass index (BMI) was calculated using the WHO classification.^16^

### Statistical analysis

Data were entered onto Research Electronic Data Capture (RedCap). At least 10% of the data was double-entered and compared, and differences were checked against the original questionnaire. The initial database containing all the entries was compared using Excel to assess an error rate within the standard allowance for acceptable error (<0.5%). Missing data participants were excluded from subgroup analyses. Data were analysed using IBM SPSS Statistics, V.27, New York., USA and R; Vienna, Austria. Descriptive statistics, including frequency distribution, were used to calculate the prevalence of outcomes and exposures. Central tendency was used to calculate weight and height as a SD. Risk factors were analysed using univariate and multivariate logistic regression. We adjusted for clusters (schools) by fitting all the exposures evaluated in the multivariate analysis into a mixed effects model analysis and assessed whether any exposure effect changed. Mixed models examine cluster-specific effects and explicitly model the random effects due to the clustering in the data.

## Results

There were 81 schools approached, and 3957 pupils from 24 schools participated; 18 were fee-paying in urban and rural areas, accommodating 23.8% and 76.2% of the population, respectively. There were 2053 (51.9% female) adolescents, and the mean and SD weight (kg) and height (cm) were 53.6±12.5 and 157.3±8.4, respectively (table 1).

**Table 1:** Characteristics of school-going adolescents aged 13–14 in KwaZulu Natal by school quintile (N=3957)

Characteristic	Non-fee paying	%	Fee-paying	%	P value*	Overall	%
	n=1173		n=2784			N=3957	
Male	568	48.4	1314	47.2	0.579	1882	47.8
Female	603	51.4	1450	52.1	0.579	2053	51.9
Urban (Durban)	0	0.0	940	33.8	<0.001	940	23.8
Rural (Richards Bay)	1173	100.0	1844	66.2	<0.001	3017	76.2
Asthma							
Lifetime asthma	266	22.7	709	25.5	0.062	975	24.6
Wheezing in the last 12 months	96	8.2	447	16.1	<0.001	543	13.7
Severe asthma	59	5.0	302	10.8	<0.001	361	9.1
Four or more attacks of wheezing in the last 12 months	26	2.2	125	4.5	0.027	151	3.8
Woken by wheezing one or more nights per week in the last 12 months	33	2.8	96	3.4	<0.001	129	3.3
Severe wheeze limiting speech to one or two words at a time in the last 12 months	37	3.2	215	7.7	0.662	252	6.4
Exercise-induced wheeze in the last 12 months	304	25.9	929	33.4	<0.001	1233	31.3
Night cough in the last 12 months	279	23.8	899	32.3	<0.001	1178	29.8
Diagnosis of asthma ever	102	8.7	410	14.7	<0.001	512	12.9
Doctor diagnosis of asthma	58	5.0	308	11.1	<0.001	366	9.2
Rhinoconjunctivitis and eczema							
Rhinitis ever	221	18.8	844	30.3	<0.001	1065	26.9
Rhinoconjunctivitis	180	15.3	540	19.4	0.003	720	18.2
Diagnosis of hay fever	90	7.7	473	17.0	0.001	563	14.2
Itchy rash ever	89	7.6	275	9.9	0.017	364	9.2
Eczema	73	6.2	207	7.4	0.174	280	7.1
Diagnosis of eczema	64	5.5	212	7.6	0.312	276	7.0
Impact of rhinoconjunctivitis							
Mild limitation	169	14.4	577	20.7	<0.001	746	18.9
Moderate-severe limitation	66	5.6	273	9.8	<0.001	339	8.6
Impact of eczema							
Less than one-night awakening per week	49	4.2	203	7.3	<0.001	252	6.4
One or more night awakening per week	40	3.4	99	3.6	<0.001	139	3.5
Asthma medication use							
Oral medication	64	5.5	257	9.2	<0.001	321	8.2
Inhaler	35	3.0	286	10.3	<0.001	321	8.1
SABA	35	3.0	223	8.0	<0.001	258	6.5
LABA	2	0.2	45	1.6	<0.001	47	1.2
ICS	2	0.2	42	1.5	<0.001	44	1.1
Combination	1	0.1	45	1.6	<0.001	46	1.2
Healthcare access							
Doctor visit last 12 months	84	7.2	399	14.3	<0.001	483	12.3
ER visit last 12 months	31	2.6	171	6.1	<0.001	202	5.1
Hospital visit last 12 months	55	4.7	211	7.6	<0.001	266	6.8
School absenteeism last 12 months	65	5.5	339	12.2	<0.001	404	10.3
Environmental exposures							
Outdoor Traffic pollution exposure	509	43.4	918	33.0	<0.001	1427	36.6
Paracetamol use in the last 12 months, n=3916	180	15.3	893	32.1	<0.001	1073	27.4
Pets	665	56.7	1504	54.0	0.160	2169	55.4
Older sibling	966	82.4	2060	74.0	0.009	3026	76.5
Younger sibling	903	77.0	1963	70.5	0.02	2866	72.4
Lifestyle exposures							
BMI Z score					0.001		
BMI<18.5: Thinness	310	26.4	530	19.0	<0.001	840	21.4
BMI≥18.5: Normal	767	65.4	1668	59.9	0.01	2435	62.0
BMI≥25: Overweight	77	6.6	425	15.3	<0.001	502	12.8
BMI≥30: Obese	15	1.3	134	4.8	<0.001	149	3.8
Mediterranean diet	728	62.1	1345	48.3	<0.001	2073	52.4
Western diet	445	37.9	1439	51.7	<0.001	1884	47.6
Current tobacco smoking (Self-reported smoking in the last 12 months)	33	2.8	84	3.0	0.72	117	3.0
Exercise according to WHO	185	15.8	383	13.8	0.126	568	14.5
Sedentary television watching	227	19.4	700	25.1	<0.02	927	23.6
Sedentary computer usage	225	19.2	629	22.6	<0.02	854	21.8

*χ^2^ test: p<0.05 was considered as significant.

BMI, body mass index; ER, emergency room; ICS, inhaled corticosteroids; LABA, long-acting beta agonist; SABA, short-acting beta agonist.

### Prevalence of asthma symptoms and asthma diagnosis by written questionnaire

Lifetime and current asthma prevalence in this population was 24.6% and 13.7%, respectively. The prevalence of severe asthma was 9.1% and measured by one or more: four or more attacks of wheeze (n=151, 3.8%), more than one night-time awakening from wheeze (n=129, 3.3%) and speech limited by wheeze (n=252, 6.4%) in the last 12 months. Only 12.9% of the population had ever had an asthma diagnosis, with the majority (71.5%) being diagnosed by a doctor. Most adolescents with current asthma (n=447/543, 82.3%) and severe asthma (n=302/361, 83.7%) were from fee-paying schools. Of those with current and severe asthma, 211 (38.9%) and 147 (40.7%) had ever had a doctor diagnosis. Of these, 72.0% and 70.7% reported using inhaled medication in the last 12 months (table 1).

### Prevalence and limitations due to rhinoconjunctivitis and eczema

The 12-month population prevalence of allergic rhinitis, rhinoconjunctivitis and eczema was 26.9%, 18.2% and 7.1%, respectively. Limitation of daily activity from nasal symptoms was reported by 27.5%, and sleep disturbance from eczema by 9.9%.

### Access to medication and healthcare utilisation

The number of adolescents who reported inhaled asthma medication use in the last 12 months (8.1%) was the same as those who used oral asthma treatment over the same duration. Inhaled controller asthma treatment use over the same period was low: only 1.1% on inhaled corticosteroids (ICS), 1.2% on long-acting beta-agonists (LABA) and 1.2% on combination treatment. The highest proportion used SABA (6.5 %). Adolescents in fee-paying schools had significantly more use of healthcare facilities with more doctor visits (14.3% vs 7.2%), ER visits (6.1% vs 2.6%) and hospital visits (7.6% vs 4.7%) in the last 12 months compared with those attending non-fee-paying schools. Adolescents from non-fee-paying schools reported less symptom-related school absenteeism (5.5% vs 12.2%) than those from fee-paying schools.

Environmental and lifestyle exposures varied across school quintiles with higher traffic pollution exposure (43.4% vs 33.0%), less overweight (6.6% vs 15.3%), less self-reported smoking (2.8% vs 3.1%), consumption of a more Mediterranean diet (62.1% vs 48.3%) and less sedentary television watching (19.4% vs 25.1%) in non-fee-paying schools compared with fee-paying schools (table 1).

Fewer adolescents (10.9%) from non-fee-paying schools reported inhaler use in the past 12 months. A minority, 13.4% of adolescents with severe asthma symptoms and no diagnosis, reported using inhalers. Of those who reported inhaler use (N=321), 30 did not indicate the type on follow-up questioning. SABAs (80.4%) were more commonly used than ICS (13.7 %). SABA was used ‘when needed’ by 81.8%, compared with ‘in short courses’ and ‘every day’ by 11.2% and 7.0%, respectively. ICS was used ‘only when needed’ by 63.6% compared with ‘in short courses’ and ‘every day’ by 22.7% and 13.6%, respectively (table 2). The majority used oral medicines: SABA syrups (7.7%), mast cell stabilisers (2.7%), theophylline (2.5%) and prednisone (1.9%) (online supplemental table A).

10.1136/bmjresp-2022-001498.supp1 Supplementary data

**Table 2:** Comparison of asthma inhaler medication use between adolescents from non-fee-paying schools and fee-paying schools in KwaZulu Natal in the last 12 months (N=321)

	Fee-paying	%	Non-fee paying	%	P value*	Total	%
Respondents who used any inhaler medicine†	286	89.1	35	10.9	<0001	321	100
SABA	223	86.4	35	13.6	0.516	258	80.4
Only when needed	181	81.2	30	85.7		211	81.8
In short courses	27	12.1	2	5.7		29	11.2
Every day	15	6.7	3	8.6		18	7.0
LABA	45	95.7	2	4.3	0.601	47	14.6
Only when needed	27	60.0	1	50.0		28	59.6
In short courses	10	22.2	1	50.0		11	23.4
Every day	8	17.8	0	0.0		8	17.0
ICS	42	95.5	2	4.5	0.132	44	13.7
Only when needed	28	66.7	0	0.0		28	63.6
In short courses	9	21.4	1	50.0		10	22.7
Every day	5	11.9	1	50.0		6	13.6
Combination	45	97.8	1	2.2	0.033	46	14.3
Only when needed	31	68.9	0	0.0		31	67.4
In short courses	9	20.0	0	0.0		9	19.6
Every day	5	11.1	1	100.0		6	13.0

*χ^2^ test: p<0.05 was considered as significant.

†n=30 did not indicate the type of inhaler used on follow-up questioning.

ICS, inhaled corticosteroids; LABA, long-acting beta agonist; SABA, short-acting beta-agonist.

The proportion of adolescents who visited the ER (6.3 vs 4.1%, p=0.002) and hospital (8.2 vs 5.4%; p<0.001) at least once in the last 12 months for uncontrolled respiratory symptoms was significantly higher in those who consumed a more Western diet compared with those who consumed a more Mediterranean diet. More children on a primarily western diet had exercise-induced wheeze (33.1 vs 29.7%; p=0.023) and cough at night (33.2 vs 27.2%; p<0.001) compared with adolescents on a primarily ‘Mediterranean’ diet (table 3).

**Table 3:** Asthma outcomes of adolescents aged 13–14 who consume primarily a Western versus a Mediterranean diet

Indicators	Mediterranean diet (%)		Western diet (%)		P value*	Overall (%)	
	n=2073		n=1884			N=3957	
Doctor visit last 12 months	236	11.4	247	13.2	0.093	483	12.3
ER visit last 12 months	85	4.1	117	6.3	0.002	202	5.1
Hospital visit last 12 months	112	5.4	154	8.2	<0.001	266	6.8
School absenteeism last 12 months	209	10.1	195	10.4	0.751	404	10.3
Exercise-induced wheeze in the past 12 months	614	29.7	619	33.1	0.023	1233	31.3
Cough at night in the past 12 months	559	27.2	619	33.2	<0.001	1178	30.1

*χ^2^ test: p<0.05 was considered as significant.

ER, emergency room.

### Risk factors for severe asthma

The odds of severe asthma were significantly increased in fee-paying school participants (1.78; (1.27 to 2.48); p=0.001). Lifestyle and environmental factors that were associated with severe asthma included; BMI z score classification as overweight (1.60; (1.15 to 2.22); p=0.005), sedentary television watching (1.42; (1.08 to 1.88); p=0.013), current tobacco smoking (2.06; (1.15 to 3.68); p=0.015) and traffic pollution exposure (1.42; (1.11 to 1.82); p=0.005). Rhinoconjunctivitis (3.62; (2.80 to 4.67); p<0.001) and eczema (2.24; (1.59 to 3.14); p<0.001) significantly increased odds for severe asthma (table 4). In the mixed model analysis, urban residence increased the odds of severe asthma (1.59; (0.34 to 2.16); p=0.03) (online supplemental table B).

**Table 4:** Univariate and multivariate analysis of factors associated with severe asthma in adolescents

Risk factor	Severe asthma		No severe asthma		Univariate analysis				Multivariate analysis			
					95% CI			P value*		95% CI		P value*
	N=361	%	N=3596	%	OR	Lower	Upper	Sign.	AOR	Lower	Upper	Sign.
Urban setting	122	33.8	818	22.7	0.577	0.458	0.727	<0.001	1.215	0.919	1.606	0.171
Fee-paying quintile	302	83.7	2482	69.0	2.297	1.723	3.064	<0.001	1.775	1.271	2.479	0.001
Sex (female)	212	59.1	1841	51.5	1.359	1.090	1.694	0.006	0.853	0.664	1.097	0.216
BMI z-score: Obese	24	6.7	125	3.5	2.181	1.376	3.457	0.001	1.524	0.904	2.570	0.114
BMI z-score: Overweight	69	19.3	436	12.2	1.798	1.342	2.409	<0.001	1.597	1.148	2.223	0.005
BMI z-score: Thin	67	18.8	771	21.6	0.987	0.739	1.318	0.930	1.112	0.807	1.531	0.517
Western diet	167	46.3	1906	53.0	1.311	1.055	1.629	0.014	0.945	0.739	1.210	0.655
Exercise according to WHO	81	22.7	487	13.6	1.859	1.426	2.424	<0.001	1.521	1.123	2.060	0.007
Sedentary television watching	135	37.6	792	22.1	2.119	1.687	2.660	<0.001	1.422	1.077	1.878	0.013
Sedentary computer use	109	30.7	745	20.9	1.675	1.319	2.129	<0.001	1.118	0.835	1.497	0.453
Current tobacco smoking	22	6.2	95	2.7	2.418	1.501	3.897	<0.001	2.059	1.152	3.679	0.015
Other types of smoking	39	11.1	241	6.8	1.710	1.196	2.446	0.003	0.898	0.578	1.394	0.630
Traffic pollution	167	47.3	1260	35.6	1.628	1.306	2.028	<0.001	1.423	1.111	1.822	0.005
Pets	219	61.2	1950	54.8	1.298	1.039	1.622	0.021	1.253	0.978	1.606	0.074
Paracetamol>1/month in last 12 months	162	44.9	911	25.3	2.469	1.977	3.084	<0.001	1.615	1.253	2.081	<0.001
Older sibling	254	70.4	2772	77	0.815	0.621	1.069	0.139				
Younger sibling	256	75.5	2610	75.1	0.976	0.753	1.265	0.854				
Rhinoconjunctivitis	171	47.4	549	15.3	4.995	3.986	6.260	<0.001	3.618	2.804	4.669	<0.001
Eczema	70	19.4	210	5.8	3.879	2.885	5.214	<0.001	2.236	1.591	3.143	<0.001

*P<0.05 was considered as significant.

BMI, body mass index.

Risk factors for current asthma matched those of severe asthma online supplemental table C. In adolescents with severe asthma symptoms, having a younger sibling was the only associated risk factor increasing the odds of lack of doctor diagnosis by 1.7-fold (table 5).

**Table 5:** Univariate analysis of factors associated with lack of diagnosis in adolescents with severe asthma in Durban, KwaZulu Natal (N=361)

Risk factors	Severe asthma without a diagnosis		Severe asthma with a diagnosis		Univariate analysis			
	n=214	%	n=147	%	OR	Lower 95% CI	Upper 95% CI	P value sign.*
Setting: Rural	74	34.6	48	32.7	1.090	0.698	1.702	0.704
Quintile: Fee-paying	176	82.2	126	85.7	0.772	0.432	1.379	0.382
Female	132	62.3	80	54.4	1.382	0.901	2.118	0.138
BMI z-score: Obese	14	6.6	10	6.8	1.019	0.432	2.407	0.965
BMI z-score: Overweight	43	20.4	26	17.8	1.204	0.686	2.115	0.518
BMI z-score: Thinness	40	19.0	27	18.5	1.079	0.614	1.896	0.793
Western diet	111	51.9	83	56.5	0.831	0.545	1.267	0.390
Exercise according to WHO	46	21.7	35	24.1	0.871	0.528	1.438	0.589
Sedentary television watching	84	39.4	51	34.9	1.213	0.783	1.878	0.387
Sedentary computer use	68	32.5	41	28.1	1.235	0.778	1.962	0.371
Current tobacco smoking	13	6.3	9	6.2	1.015	0.422	2.441	0.974
Other types of smoking	19	9.2	20	13.7	0.640	0.328	1.248	0.190
Traffic pollution	102	48.6	65	45.5	1.133	0.740	1.735	0.565
Pets	134	63.5	85	57.8	1.269	0.825	1.953	0.278
Paracetamol more than once a month in last 12 months	91	44.2	71	48.3	0.847	0.554	1.295	0.443
Older sibling	152	78.4	102	75.6	1.171	0.696	1.970	0.552
Younger sibling	158	79.4	98	70.0	1.652	1.003	2.719	0.049
Rhinoconjunctivitis	95	44.4	76	51.7	0.746	0.489	1.136	0.172
Eczema	39	18.2	31	21.1	0.834	0.492	1.412	0.499

*P<0.05 was considered as significant.

BMI, body mass index.

## Discussion

In this GAN survey of adolescents in South Africa, we found a prevalence of current wheeze (13.7%) higher than the global average (10.4%) but lower than that reported in a more urban population in Cape Town.^5^ Despite a high burden of asthma in our population, inhaled medication use, particularly with ICS, was only 1%, a finding illustrating gaps in asthma care shared by many LMICs, including India.^18^

The rural-urban difference in severe asthma seen in the mixed model analysis is in keeping with several studies in LMICs.^19^ One of these, a Ugandan study, found a strong rural-town-city risk gradient among school children aged 5–7 years.^20^ Those born in a small town or the city had an increased asthma risk compared with those born in rural areas (2.16 (1.60 to 2.92)) and (2.79 (1.79 to 4.35)), respectively. While farming and exposure to livestock have been suggested to have a protective effect on the development of asthma in HICs, this is not so pronounced in LMICs, where rapid urbanisation coupled with exposure to environmental pollution and lifestyle changes may have a dominating causal effect.^19^

Using school quintiles as a proxy for SES, we found that children from fee-paying schools had a higher asthma prevalence and more severe asthma symptoms. Although this proxy reflects a population-level measure, our findings represent a different perspective on the influence of economic status on asthma prevalence and severity. Urban lifestyle factors like diet, obesity and traffic pollution may increase with improving SES and are emerging targets for interventions that would impact asthma outcomes.^7 21^

Providing access to ICS is key to improving the quality of care for asthma in LMICs, where affordable drugs would reduce the burden on health systems and people affected by asthma.^2^ Despite the socioeconomic differences in this population, access to diagnosis and treatment remains poor overall. Even with better access to inhaled therapy in more affluent children, its incorrect use may negate its effect. A notable difference between socioeconomic groups was seen in health-seeking behaviour, with the underprivileged having less access to doctors and emergency and healthcare facilities. Similar to an asthma cohort in Zimbabwe, this could reflect poor access due to limited resources.^22^ However, their preference for alternate care in managing uncontrolled asthma symptoms, including traditional healer visits and use of alternative treatment was highlighted and requires further probing in South Africa and other LMICs.^22 23^

Furthermore, in adolescents with severe asthma, those with younger siblings were more likely to lack diagnosis. A possible explanation is that viral infections, more frequent in younger siblings, may exacerbate their adolescent sibling’s asthma symptoms, who may repeatedly and suboptimally be treated for the viral illness rather than diagnosed with asthma. Also, some adolescents with severe asthma symptoms received inhalers but not a diagnosis. In this setting, diagnosis of asthma and adherence to asthma guidelines is a critical gap in asthma management, as ICS and diagnostic guidelines are available in the primary care setting in South Africa.^24^ Strategies to increase access to basic effective asthma care, including non-physician-led optimisation of inhaled medicines and individualised education, are feasible and impactful in LMICs.^25^

The recent key step change of asthma management to include ICS whenever a SABA is taken or ICS-LABA with symptoms remains a challenge in LMICs.^3 26^ Similar to our South African cohort, SABA overuse/overreliance is still commonly reported in sub-Saharan Africa (sSA).^3 27^ Of concern is the high proportion of adolescents who are still prescribed oral asthma treatments against local guidelines.^24^ Studies across Africa show that people with asthma symptoms generally prefer oral medication and are reluctant to use inhaled asthma treatment.^28 29^ Strategies leading to better adherence and asthma control, including education, can dispel myths, reduce stigma and improve perceptions and attitudes around asthma and asthma treatments.^25^ Furthermore, the lack of clinical trial evidence from LMICs perpetuates their inability to appropriately inform decision-making and asthma healthcare.^18^

Exposure to outdoor pollution was significantly associated with asthma symptoms in the study, consistent with other African studies.^8 30^ However, these could be limited by biased traffic reporting, where those who may have symptoms and are aware of the risks of traffic pollution may report increased truck exposure. There is little data on air pollution and its effects on asthma in our setting. In Durban, 52% of children living in a highly polluted factory district reported asthma symptoms in the preceding 12 months.^31^ Similarly, self-reported rates of wheeze in the last 12 months (37%) in school-going children residing in a highly polluted area in Durban were strongly correlated with school absence.^32^ Increasing urbanisation, poor air quality and traffic pollution in emerging cities have been directly correlated to respiratory illnesses, including chronic obstructive pulmonary disease (COPD) in adults and asthma symptoms in children.^30 33^ An increasing number of air pollution interventional studies conducted in LMICs mainly address indoor biomass exposures, and these have not shown efficacy in reducing morbidity and mortality in children.^34 35^ Moreover, many air pollution studies rely on macroenvironment markers of exposure and give little insight into precise microenvironment measurements of pollutants such as black carbon and particulate matter.^36^

Self-reported smoking was similar across affluent and non-affluent schools and reflected previous literature as a significant risk factor for current and severe asthma.^37^ Environmental tobacco smoking (ETS), a well-established cause and trigger for asthma, is also a leading risk factor for COPD, a major cause of morbidity and mortality in sSA.^2^ Furthermore, ETS exposure in asthmatic individuals has been associated with worse lung function, more asthma exacerbations and greater usage of emergency care services.^38^ There is a need to understand the determinants of adolescent smoking in this setting to enhance antitobacco messaging and policy making, particularly as smoking increases the odds of COPD by twofold.^2^

In our cohort, higher SES and urban lifestyle appear to be associated with severe asthma and higher morbidity. Similarly, American inner-city children with asthma were significantly more overweight than controls.^39 40^ Interestingly, adolescents in this study who were primarily on a western diet significantly contributed to most hospital visits for respiratory symptoms. Contrasting a recent South African cohort, a Western diet did not increase the likelihood of current or severe asthma in our population.^41^ However, this observation is in keeping with a systematic review, where although no relation between risk of incidence or prevalence of asthma was found in 70 000 individuals, there was an association between severe asthma symptoms and a Western diet.^42^

In our cohort, the likelihood of current and severe asthma increased by at least twofold and threefold with eczema and rhinoconjunctivitis. While atopy-associated asthma is more prevalent in countries with the highest economic status, a rural-urban difference has been noted in Africa with increased sensitisation in urban children.^43^

The current study is limited as a questionnaire-based survey that relies on self-reporting (which may be influenced by recall and language) instead of objective markers for confirming asthma and exposures. However, the GAN questionnaire is a standardised and globally implemented instrument based on the methodology of ISAAC, which has concurrent and predictive validity.^12^ Indoor exposures including pets, particularly birds contribute to symptoms of eczema, rhinitis and allergic asthma. However, the GAN questionnaire elicits the presence of cats and dogs only. Similarly, the GAN questionnaire does not elicit health seeking practices including use of alternative treatments and traditional healers. As no additional tool was used, this information cannot be included in our study and is a limitation. Furthermore, the epidemiological nature of the study precludes our ability to identify causal relationships with certainty. However, these are useful for establishing preliminary evidence for a causal relationship between asthma, severe asthma and atopy. Our study had strengths as the study participants were representative of the population of school-going children in KZN who are primarily rural. Despite a disparity in number, participants from rural and urban areas were represented in the study.

In conclusion, asthma is common in South Africa. Severe asthma symptoms are underdiagnosed and associated with atopy, environmental, lifestyle and dietary factors. Where there is a diagnosis, inhaler treatment remains underused with SABA overreliance. Solutions geared at long-term follow-up focusing on asthma education and issuing appropriate medication for symptom control may be beneficial in LMICs. A World Health Assembly resolution to ensure access to asthma medicines, such as that with other non-communicable diseases, including Diabetes Mellitus, will ensure all asthmatics access to affordable, quality-assured ICS.

## Data Availability

Data are available on reasonable request. Data analysis is ongoing for PhD thesis of RMp. Datasets will be made publicly available on completion of her PhD.
