# Supplementary material for: Prevalence, severity and risk factors for asthma in school-going adolescents in KwaZulu Natal, South Africa
Source: BMJ Open Respir Res. 2023 May 16;10(1):e001498. doi: 10.1136/bmjresp-2022-001498 (PMC10193066; doi:10.1136/bmjresp-2022-001498)
Supplement: Supplementary data [file bmjresp-2022-001498supp001.pdf]

Supplementary Table A: Oral asthma medications and frequency of use by adolescents in KwaZulu Natal (N=3957).

Frequency	No. of adolescents	Usage by population (%)	Usage by drug (%)
Prednisone			
Only when needed	57	1.4	74.0
In short courses	15	0.4	19.5
Everyday	5	0.1	6.5
Total	77	1.9	100
Short acting beta agonist			
Only when needed	232	5.9	76.6
In short courses	54	1.4	17.8
Everyday	17	0.4	5.6
Total	303	7.7	100.0
Theophylline			
Only when needed	69	1.7	71.1
In short courses	19	0.5	19.6
Everyday	9	0.2	9.3
Total	97	2.5	100.0
Mast cell stabilizers			
Only when needed	80	2.0	74.1
In short courses	22	0.6	20.4

Everyday	6	0.2	5.6
Total	108	2.7	100.0

Supplementary Table B: Mixed models effect analysis of factors associated with severe asthma in 13-14-year-old adolescents in Durban, KwaZulu Natal (KZN).

Effect	NA	Term	Estimate	std error	statistic	*p value
Fixed	NA	Intercept	3.233	5.939	0.639	0.523
Fixed	NA	Urban	1.593	0.344	2.161	0.031
Fixed	NA	Non-fee-paying school quintile	0.575	0.133	-2.391	0.017
Fixed	NA	Age	0.723	0.098	-2.399	0.016
Fixed	NA	BMI z score Obese	1.623	0.450	1.744	0.081
Fixed	NA	BMI z score Overweight	1.622	0.289	2.709	0.007
Fixed	NA	BMI z score Thinness	1.024	0.182	0.136	0.892
Fixed	NA	Female	1.087	0.147	0.617	0.537
Fixed	NA	Rhinoconjunctivitis	3.640	0.504	9.331	<0.001
Fixed	NA	Eczema	2.189	0.411	4.172	<0.001
Fixed	NA	Exercise according to WHO	1.429	0.238	2.142	0.032
Fixed	NA	Sedentary television watching	1.472	0.222	2.590	0.009
Fixed	NA	Sedentary computer use	1.182	0.185	1.070	0.284
Fixed	NA	No Older sibling	1.004	0.159	0.023	0.817
Fixed	NA	No Younger sibling	0.953	0.144	-0.316	0.751
Fixed	NA	Western diet	0.917	0.123	-0.644	0.519
Fixed	NA	Paracetamol more than once a month in last 12 months	1.566	0.216	3.255	0.001
Fixed	NA	Pets	1.215	0.161	1.468	0.142
Fixed	NA	Current Smoker	2.158	0.699	2.376	0.018
Fixed	NA	Other types of smoking	0.841	0.209	-0.699	0.484
Fixed	NA	Traffic pollution (not exposed)	0.701	0.094	-2.646	0.008
ran_pars school	NA	sd_Intercept	0.264	NA	NA	

BMI = Body mass index; WHO = World Health Organization; *p-value < 0,05 was considered as significant

Supplementary Table C: Univariate and multivariate analysis of factors associated with current wheeze in adolescents.

Risk Factors	Current wheeze		No severe wheeze		Univariate analysis				Multivariate analysis			
						95% CI		*p value		95% CI		*p value
	n=543	%	n=3414	%	OR	Lower	Upper	Sign.	AOR	Lower	Upper	Sign.
Setting Rural	384	70.7	2633	77.1	0.716	0.586	0.876	0.001	0.976	0.770	1.236	0.841
Fee-paying school quintile	447	82.3	2337	68.5	2.146	1.702	2.706	<0.001	1.951	1.492	2.552	0.000
Female	315	58.6	1738	51.2	1.348	1.121	1.621	0.001	1.147	0.934	1.409	0.191
BMI z-score obese	34	6.3	115	3.4	2.181	1.376	3.457	0.001	1.287	0.818	2.026	0.276
BMI z-score overweight	86	16.0	419	12.4	1.798	1.342	2.409	0.000	1.086	0.814	1.449	0.576
BMI z-score thinness	88	16.4	750	22.1	.987	.739	1.318	0.930	0.735	0.558	0.969	0.029
Western diet	262	48.3	1811	53.1	1.212	1.011	1.453	0.037	0.970	0.792	1.188	0.771
Exercise according to WHO	101	18.8	467	13.8	1.451	1.144	1.84	0.002	1.308	1.003	1.706	0.047
Sedentary television watching	168	31.2	759	22.3	1.573	1.289	1.920	<0.001	1.199	0.944	1.523	0.138
Sedentary computer use	138	25.8	716	21.2	1.294	1.049	1.598	0.016	0.968	0.752	1.246	0.801
Current tobacco smoking	28	5.2	89	2.6	2.034	1.316	3.141	0.001	1.715	1.028	2.862	0.039
Other types of smoking	49	9.2	231	6.9	1.381	1	1.908	0.05	0.958	0.661	1.389	0.822
Traffic pollution	219	41.2	1208	35.9	1.249	1.037	1.505	0.019	1.168	0.949	1.436	0.142
Pets	327	60.8	1842	54.5	1.291	1.072	1.555	0.007	1.268	1.034	1.553	0.022
Paracetamol more than once a month in last 12 months	222	41.4	851	25.2	2.101	1.74	2.537	<0.001	1.528	1.235	1.891	0.000
Older sibling	399	79.2	2627	80.5	0.921	0.731	1.162	0.489				
Younger sibling	392	76.0	2474	75.0	0.947	0.763	1.177	0.625				
Rhinoconjunctivitis	217	40.0	503	14.7	3.852	3.166	4.687	<0.001	3.032	2.437	3.773	0.000
Eczema	87	16.0	193	5.7	3.184	2.427	4.177	<0.001	2.059	1.515	2.797	0.000

BMI = Body mass index; WHO = World Health Organization; *p-value < 0,05 was considered as significant
